# Dopamine Modulates Option Generation for Behavior

**DOI:** 10.1016/j.cub.2018.03.069

**Published:** 2018-05-21

**Authors:** Yuen-Siang Ang, Sanjay Manohar, Olivia Plant, Annika Kienast, Campbell Le Heron, Kinan Muhammed, Michele Hu, Masud Husain

**Affiliations:** 1 Department of Experimental Psychology, University of Oxford, Anna Watts Building, Radcliffe Observatory, Woodstock Road, Oxford OX2 6GG, UK; 2 Nuffield Department of Clinical Neurosciences, University of Oxford, Level 6, West Wing, John Radcliffe Hospital, Oxford OX3 9DU, UK

**Keywords:** dopamine, option generation, decision making, Parkinson’s disease, creativity, apathy, cabergoline

## Abstract

Animals make innumerable decisions every day, each of which involves evaluating potential options for action. But how are options generated? Although much is now known about decision making when a fixed set of potential options is provided, surprisingly little progress has been made on self-generated options. Some researchers have proposed that such abilities might be modulated by dopamine. Here, we used a new measure of option generation that is quantitative, objective, and culture fair to investigate how humans generate different behavioral options. Participants were asked to draw as many different paths (options) as they could between two points within a fixed time. Healthy individuals (n = 96) exhibited a trade-off between uniqueness (how individually different their options were) and fluency (number of options), generating either many similar or few unique options. To assess influence of dopamine, we first examined patients with Parkinson’s disease (n = 35) ON and OFF their dopaminergic medication and compared them to elderly healthy controls (n = 34). Then we conducted a double-blind, placebo-controlled crossover study of the D2 agonist cabergoline in healthy older people (n = 29). Across both studies, dopamine increased fluency but diminished overall uniqueness of options generated, due to the effect of fluency trading off with uniqueness. Crucially, however, when this trade-off was corrected for, dopamine was found to increase uniqueness for any given fluency. Three carefully designed control studies showed that performance on our option-generation task was not related to executing movements, planning actions, or selecting between generated options. These findings show that dopamine plays an important role in modulating option generation.

## Highlights

• Trade-off between fluency and uniqueness exists when generating behavioral options
• Dopamine improved fluency but diminished overall uniqueness due to this trade-off
• Correcting for trade-off revealed dopamine increased uniqueness for a given fluency
• Results cannot be explained by motor execution, action planning, or option selection

## Graphical Abstract

**Figure:**
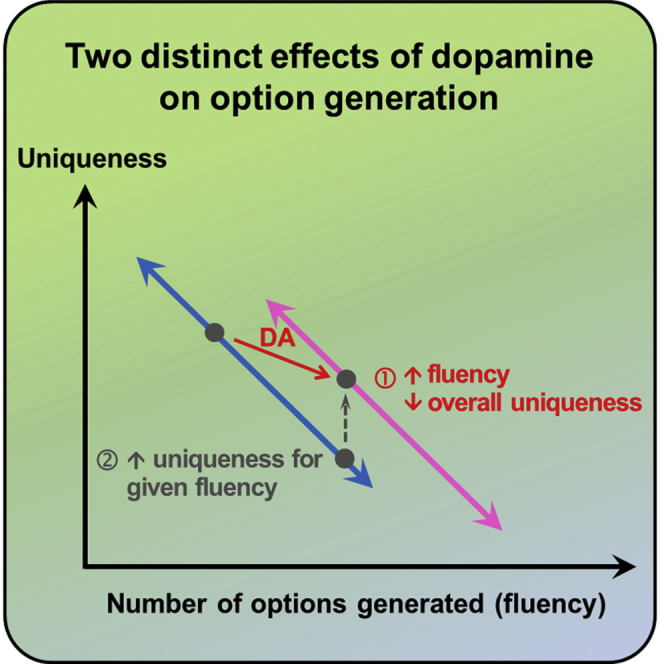

## Teaser

Little is known about how humans generate options for behavior. Ang et al. devised a quantitative and objective measure of option generation and demonstrated, using patients with Parkinson’s disease and pharmacological manipulation in the healthy population, that dopamine plays an important role in modulating this process during decision making.

## Introduction

The neuroscience of decision making has focused on decisions between options provided by the experimenter [1], but it is increasingly recognized that such forced choice scenarios might be limited in ecological validity. As a result, some recent research has instead shifted toward decisions about exploiting current environments versus exploring new options—termed foraging—which is considered more natural [2]. But this work still assumes that options for behavior are already visible in the environment, which is not always the case in the real world [3, 4]. How we self-generate options for behavior remains poorly understood. Two different domains of research, executive control and creative cognition, suggest a key role for the prefrontal cortex in generating numerous options for behavior (fluency) [5, 6] and in the uniqueness of those options [7, 8, 9, 10, 11, 12, 13]. Prefrontal cortex has long been identified to play an important role in executive functions crucial for the initiation and sustaining of responses [5, 6, 14]. Neuroimaging and lesion studies have implicated the frontal lobe in verbal and nonverbal tests of fluency that require the production of as many responses as possible in a fixed time limit (see MacPherson et al. [14] for review). The frontal cortex is also considered to play a key role in creativity, with evidence from neuroimaging [7, 8], electrophysiological [9], and patient studies [10, 11] lending support to this view. Furthermore, focal lesions of the frontal lobe impair performance across various measures of creativity [12, 13].

However, the neurochemical modulation of these two aspects of option generation and how they might relate to each other remains unknown. It has been previously speculated that dopamine may be involved in option generation [15]. Based on several lines of research, e.g., genetics [16, 17], neuroimaging [18, 19], and patient [20, 21] studies, a related theory in creative cognition also proposed recently that persistence in generating many ideas and flexibility in producing novel ideas may be modulated by dopamine [22]. However, these premises have never been tested, and thus, no direct supporting evidence exists. Here, we hypothesize that dopamine plays a specific role in improving fluency of option generation and increasing the uniqueness of these options. Further, if option generation is indeed associated with individual differences in dopaminergic tone, we predicted that it would relate to traits such as motivation [15].

These questions are empirically challenging to investigate. Some previous studies of option generation have focused on decision-making settings that are very specific, e.g., chess problems [23] or sports [24]. These complex scenarios have the advantage of affording a wide range of possible options and might consequently be considered to be more relevant to real-life option generation, where the option space is wide. However, in these domains, individuals may still be limited by their understanding and familiarity with the activity being tested. Alternatively, executive tests of fluency [14, 25, 26, 27] and fluency-based creativity tests (e.g., alternative uses test; see [22]) can be used, but these tasks involve generating discrete outputs, such as words, and often require subjective assessments of novelty. Moreover, the paradigms often involve searching a semantic space, and so performance is strongly biased by an individual’s educational and cultural background. It might also be affected by linguistic ability, prospective or counterfactual thinking, and working memory. To overcome these limitations, we developed a novel measure of option generation that is simple and quantitative, objective, and culture-free. Whereas it does not provide assessment of different types of possible choices, it is relatively unconstrained, allowing people to generate on their own different options to solve a simple problem that can be understood without extensive experience required.

**Figure 1:**
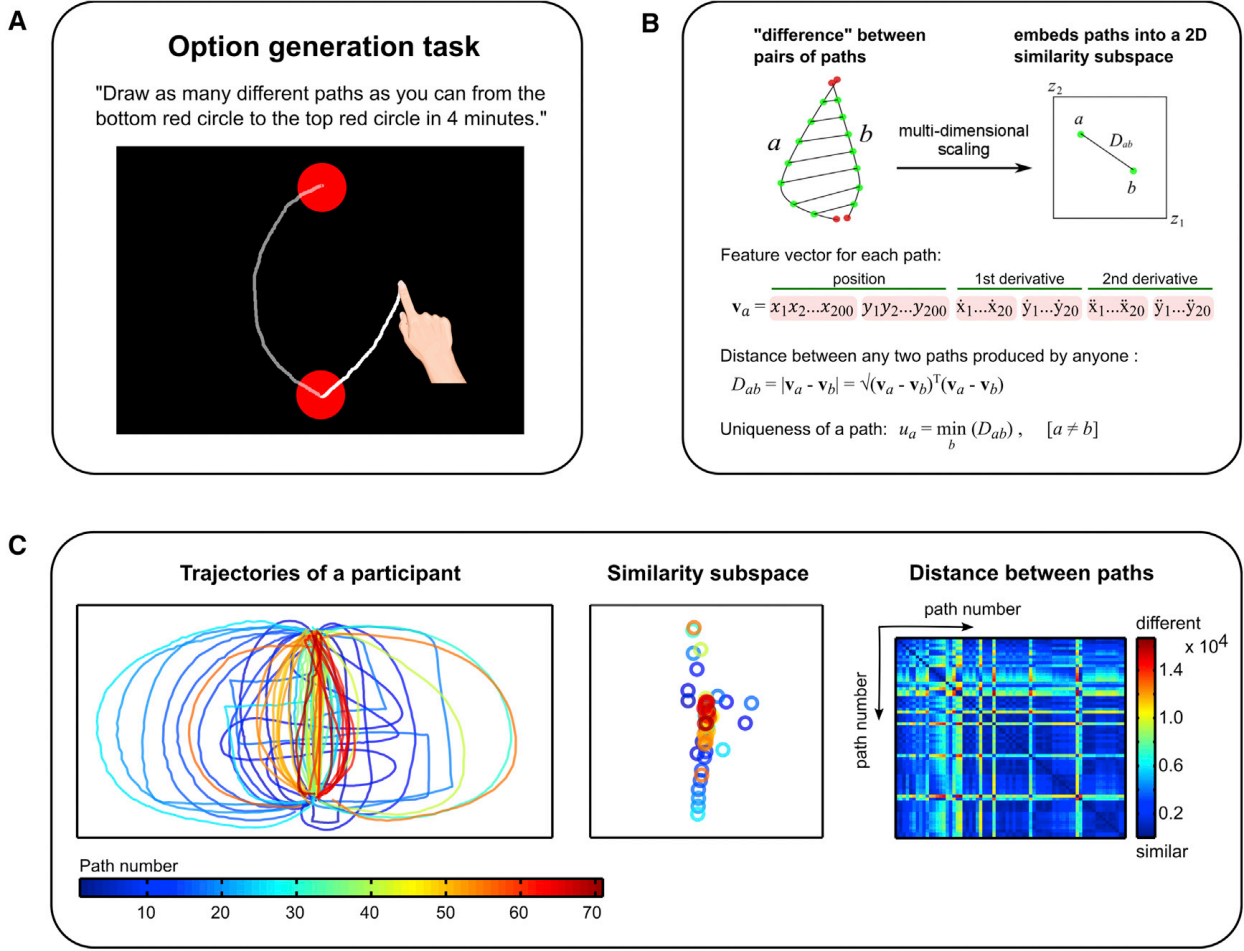
Option-Generation Task (A) The option-generation task required participants to draw, in 4 min, as many different paths as possible from the start point (bottom circle) to the end goal (top circle). Paths appeared as participants drew them and remained displayed on the touchscreen during the task so that participants did not have to remember them. (B) To quantify uniqueness, each path was first parameterized by 200 equally spaced points along its length. The “difference” between any two paths is then taken as the sum of the distance between corresponding points. This distance metric also includes the first and second derivatives in order to better account for curvatures in trajectories. Uniqueness of a path is then defined as the “distance” between it and the most similar path generated (by any participant in the three studies of this paper). Distances between a participant’s paths were also projected into a 2-dimensional subspace using multidimensional scaling to visualize how individuals explored the space of possible paths by treating each generated path as a point. Points that are closer together indicate more similar paths and vice versa. (C) Illustrations of the trajectories of the 69 paths generated by one participant (left), his corresponding points in 2-dimensional similarity subspace after multidimensional scaling (middle), and the pairwise distance matrix (right). See also Figure S1.

Participants were given a time limit of four minutes to draw on a touchscreen computer as many different paths as they could between two vertically aligned, fixed points (see STAR Methods; Figure 1A). This allowed us to extract movement and timing parameters, thereby providing an opportunity to evaluate both the uniqueness and diversity of paths generated using objective metrics.

To quantify uniqueness, we created an index of similarity between pairs of paths (Figure 1B). Each path was parameterized by 200 points equally spaced along its length, and the distance between a pair of lines drawn on the touchscreen was computed as the sum of distances between pairs of corresponding points. Lines were reflected about the midline such that mirror image paths were considered as similar to each other, and the first and second derivatives were included in the distance metric to better account for differences in the shapes of the curves. This allowed us to measure “effective distance” between each pair of paths drawn. For every path produced by each participant, uniqueness was defined as the “distance” between it and the most similar path produced by all other participants in the three studies of this paper.

To quantify diversity, the distances between a participant’s paths were projected onto a 2-dimensional subspace using multidimensional scaling to visualize how they explored the path space (Figures 1B and 1C). An individual was more explorative, i.e., produced greater variation in generated paths, if the paths (each represented by one point in the dissimilarity space) were more dispersed. We approximated this by calculating the area of the convex hull covering each participant’s paths projected in the 2-dimensional subspace (see STAR Methods for details).

## Results and Discussion

**Figure 2:**
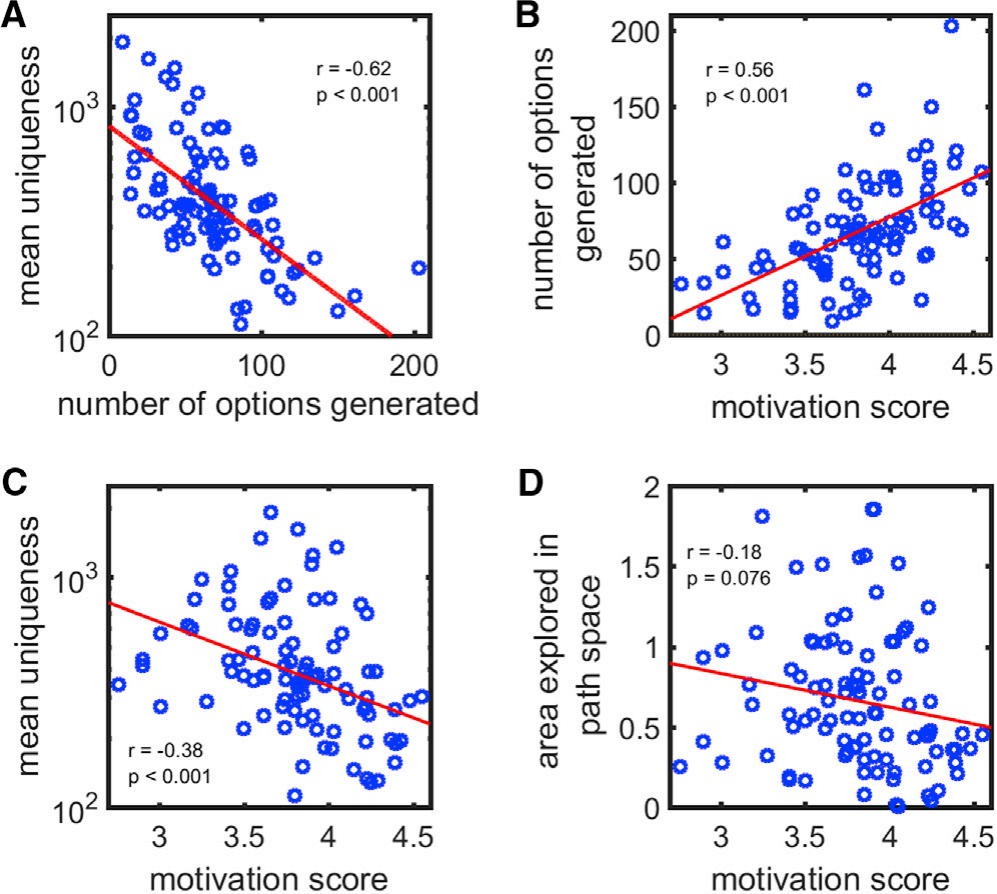
Results from the Option-Generation Task in Healthy People Each participant is represented by one point. (A) A scatterplot of the mean uniqueness against the number of paths generated, or fluency, revealed a uniqueness-fluency trade-off. Participants tended to either generate a few unique paths or many similar paths. (B) The number of paths generated on this task correlated positively with the level of motivation as indexed by a self-report measure. Thus, the more apathetic an individual, the fewer paths they were likely to generate. (C) The mean uniqueness of an individual’s paths is plotted against their motivation score. Motivated individuals generated less unique paths whereas apathetic people produced more unique paths. (D) Exploration was quantified by treating each generated path as a point in the individual’s 2-dimensional uniqueness subspace (see Figure 1C). The total “area” of different paths explored was defined as the area of a polygon surrounding all the path points (i.e., the convex hull). There was a trending negative correlation between explored area and motivation. This suggests a greater diversity of paths explored in apathetic individuals despite the fact that they generated a smaller number of paths, although that must be interpreted with caution.

We first administered this task to a group of 96 young and elderly healthy individuals (study 1; see STAR Methods). Because there was no difference in fluency (*t*(94) = 0.97; p > 0.05), uniqueness (*t*(94) = 0.19; p > 0.05), and diversity (*t*(94) = 0.57; p > 0.05) between the two age groups, the data for both cohorts were combined and presented together. Intriguingly, healthy people displayed a trade-off between uniqueness and fluency. They tended to either produce many similar paths or came up with fewer unique paths (r = −0.62; p < 0.001; BF_10_ > 100; Figure 2A; see Figure S1 for more example trajectories). Individual differences in motivation level as indexed by an independent, self-report measure (STAR Methods) were differentially associated with uniqueness and fluency. Motivation scaled positively with the number of paths generated (r = 0.56; p < 0.001; BF_10_ > 100; Figure 2B) but negatively with overall uniqueness (r = −0.38; p < 0.001; BF_10_ > 100; Figure 2C). This suggests that motivation level might influence performance along the uniqueness-fluency spectrum. Less motivated—or apathetic—individuals appeared to be biased toward generating fewer paths *but* crucially with greater uniqueness, whereas motivated individuals trade uniqueness to produce more paths.

There was also a trend for the area explored in the 2-dimensional path subspace to correlate negatively with an individual’s level of motivation, with apathetic people covering a larger area (r = −0.18; p = 0.076; BF_10_ = 0.60; Figure 2D). Whereas this must be interpreted with caution, it suggests that, although apathetic individuals generated fewer paths, they tended to be more diverse in terms of the space of possibilities. Because fluency and uniqueness are correlated, we asked whether they independently relate to motivation. A univariate generalized linear model (GLM) demonstrated that fluency (p = 0.01), but not uniqueness (p > 0.05) or area of exploration (p > 0.05), predicted level of motivation. Fisher-transformed z tests also showed that the correlation between fluency and motivation is significantly different to that between uniqueness and motivation (z = 5.67; p < 0.001) and that between area of exploration and motivation (z = 4.94; p < 0.001).

How might the uniqueness and fluency in generating options be influenced by dopamine? To answer this question, we tested 35 patients with Parkinson’s disease (PD), a neurodegenerative disorder characterized by loss of dopaminergic neurons in the substantia nigra and with evidence of prefrontal cortical dysfunction [28, 29]. PD is also associated with a deficit in self-initiated movements [30, 31]. These patients were tested twice in two counterbalanced sessions—once ON dopaminergic medication and once after overnight withdrawal (“OFF” state). This design allowed us to compare performance when dopamine levels differ within-subject, thereby permitting inferences to be made regarding influence of dopamine. We also examined whether the effects of dopamine on option generation was influenced by pathological lack of motivation—apathy—in PD. 34 healthy age-matched controls were also recruited, of whom 18 were tested once and 16 twice (study 2; STAR Methods).

**Figure 3:**
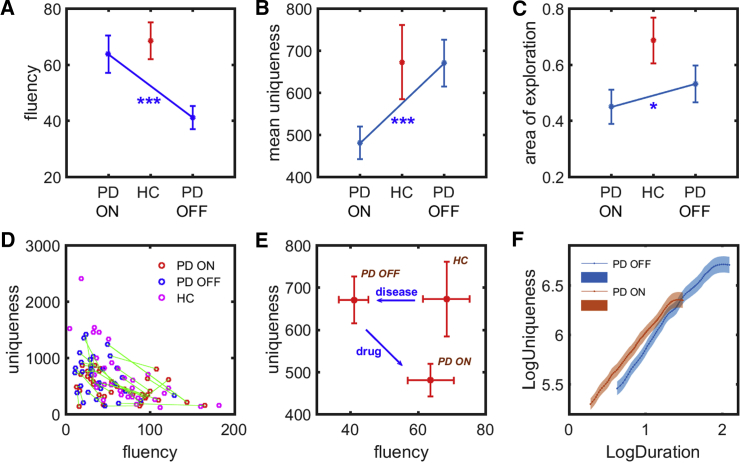
Option-Generation Task Performance in Parkinson’s Disease versus Controls (A–C) PD patients generated significantly more paths when ON compared to OFF dopamine (p < 0.001) (A). Yet, they were more unique (p < 0.001) (B) and explored a larger area in the 2-dimensional path subspace (p < 0.05) (C) when OFF their medication. Healthy age-matched controls were both fluent and creative, generating a similar number of paths to patients ON dopamine and displaying comparable uniqueness and area of exploration to patients OFF dopamine. (D) A scatterplot of each individual’s mean uniqueness of paths against their number of paths generated. The same PD patients ON and OFF dopamine are connected by green lines. There appears to be a uniqueness-fluency trade-off between generating more paths and the uniqueness of these paths. (E) A comparison of the group averages in uniqueness and fluency shows that PD reduces fluency of generation, although uniqueness is preserved. Administration of dopamine restores the number of paths generated but at the expense of reduced uniqueness. This suggests that dopamine may play an important role in modulating the balance between generating more options against producing more unique outputs. (F) Because fluency correlates with uniqueness, a linear mixed effects model was used to examine how each path’s duration (or inverse of fluency) influences uniqueness. Dopamine was found to increase the uniqueness of paths after correcting for the fact that they were faster (red line significantly higher than blue line; p = 0.005). Error bars refer to one SE. HC, healthy controls; PD, Parkinson’s disease. See also Figures S2, S3, S4, S5, and S7 and Table S3.

Paired-samples t test revealed a significant effect of drug state (ON versus OFF) on performance in our option-generation task. PD patients generated fewer paths when OFF compared to ON dopamine (*t*(34) = 4.51; p < 0.001; BF_10_ > 100; Figure 3A). But when OFF medication, they exhibited greater mean uniqueness in their generated paths (*t*(34) = −3.76; p < 0.001; BF_10_ = 47.1; Figure 3B) and were more explorative in the 2-dimensional path subspace (*t*(34) = −2.16; p < 0.05; BF_10_ = 1.4; Figure 3C). Thus, dopamine modulated the balance between uniqueness of paths and the fluency of path generation (Figure 3D). There was no significant effect of testing on repeated sessions in patients.

Because fluency correlates with uniqueness, a mixed model was used to examine the effect of each path’s duration on uniqueness, as a function of drug state and apathy. This analysis revealed that dopamine crucially increased the uniqueness of paths after correcting for the fact they were faster (*t*(3,661) = 2.81; p = 0.005), but apathy had no main effect or interaction. Thus, although the major drug effect was to produce a trade-off, with faster and less-unique paths, it also independently improved uniqueness for a given fluency (Figures 3F and S3).

Age-matched controls were both fluent and unique, generating a comparable number of paths to PD patients ON dopamine (ON: *t*(67) = −0.51, p > 0.05, BF_10_ = 0.28; OFF: *t*(67) = −3.55, p < 0.001, BF_10_ = 40.8; Figure 3A) and exhibiting comparable uniqueness to patients when OFF dopamine (ON: *t*(67) = −2.03, p < 0.05, BF_10_ = 1.40; OFF: *t*(67) = −0.03, p > 0.05, BF_10_ = 0.25; Figure 3B). Note that we used data from only the first session of controls here because no significant difference across sessions was found (Figure S3). By comparing the average uniqueness and fluency between the three groups—PD ON, PD OFF, and healthy control—it is evident that the PD state lowers the fluency of generation without affecting uniqueness. Treatment with dopamine helps to restore the number of paths generated but at the expense of uniqueness (Figure 3E). This suggests that dopamine may play an important role in modulating the balance between generating more options against producing more unique options.

One might argue against this conclusion by suggesting that the effect of dopamine on increasing fluency is simply due to improvement in the motor deficits that are characteristic of PD. Further analysis of the data suggests this is unlikely. We examined the severity of motor deficits for each patient in both the ON and OFF states using the Unified PD Rating Scale (UPDRS) and found that the difference in UPDRS motor scores between the ON and OFF states correlated strongly with that in the OFF state (r = 0.54; p = 0.001; BF_10_ = 46.8; Figure S5D). Thus patients with worse PD severity also showed greater improvement in motor symptoms when treated with dopamine. The UPDRS motor scores in the OFF state also correlated with the number of paths generated both when ON and OFF dopamine but importantly *not* with the difference in fluency between the two states, i.e., ON-OFF (ON: r = −0.36, p < 0.05, BF_10_ = 1.8; OFF: r = −0.48, p < 0.01, BF_10_ = 13.5; ON-OFF: r = 0.08, p > 0.05, BF_10_ = 0.24; Figures S5A–S5C). Thus disease severity (indexed by UPDRS score) modulated the baseline number of paths generated. However, it had no effect on dopaminergic improvement in fluency, despite strongly predicting improvement in motor UPDRS scores when ON dopaminergic drugs (difference in correlation coefficients; Fisher-transformed z = 2.24; p < 0.05). The improvement in fluency on dopamine was also not correlated with dopaminergic improvement in motor symptoms based on UPDRS, indicating that the increase in number of options generated was not due to better motor abilities (r = 0.14; p > 0.05; BF_10_ = 0.30).

Could the increased uniqueness displayed by PD patients when OFF dopamine be due to increased tremor? This is unlikely. We derived an index of tremor by combining the UPDRS resting and kinetic tremor severity scores and found no significant relationship between amount of tremor and uniqueness (Spearman ρ = 0.05; p > 0.05). Furthermore, in a separate analysis, the raw movement data were first smoothed using a moving average window size of 250 ms (*smoothdata* function in MATLAB R2017a) to remove energy around 4 Hz from tremors before computing uniqueness. The results revealed that PD patients still displayed greater uniqueness when OFF compared to ON dopamine (*t*(34) = 3.76; p < 0.001), suggesting that tremor during the OFF state did not contribute to increased uniqueness.

Next, we enquired whether dopamine might have similar effects in healthy participants. We investigated the effects of cabergoline—a dopamine D_2_ agonist—on option generation in a within-subject, double-blind, and placebo-controlled design (study 3; see STAR Methods). 29 healthy elderly individuals were tested twice in two counterbalanced sessions. They took a 1-mg cabergoline tablet on one session and an indistinguishable placebo tablet on the other.

**Figure 4:**
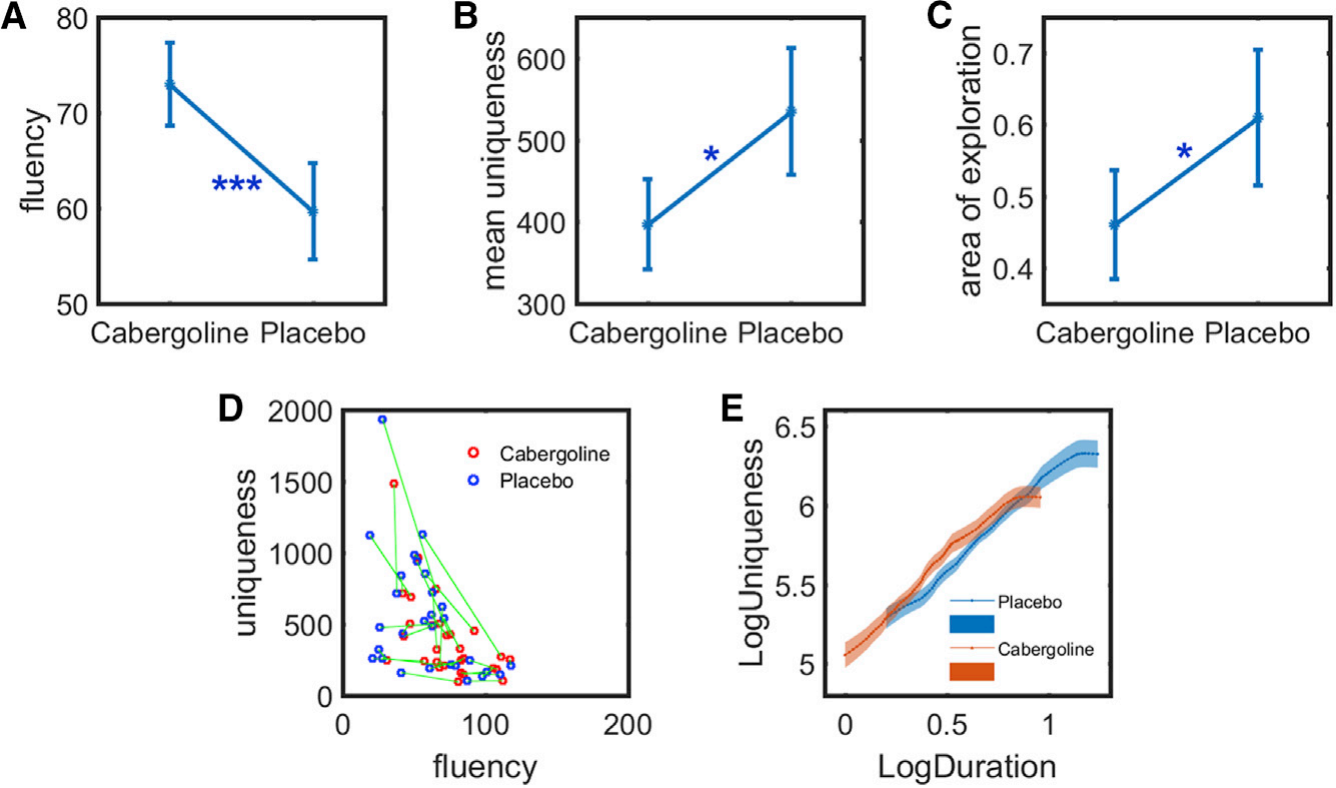
Option-Generation Task Performance in Healthy Elderly People (n = 29) on Cabergoline and Placebo (Study 3) (A–C) Healthy people generated significantly more path options when they have taken cabergoline (dopamine D_2_ agonist) compared to placebo (p < 0.001) (A). Yet they were more unique (p < 0.05) (B) and explored a larger area in the 2-dimensional path subspace (p < 0.05) (C) when they were on placebo. (D) A scatterplot of each individual’s mean uniqueness of paths against their number of paths generated. The same individual when on cabergoline and placebo are connected by green lines. (E) A mixed model applied to account for the correlation between fluency and uniqueness found that dopamine increases the uniqueness of paths for a given fluency (p < 0.001), which is consistent with what we have found from Parkinson’s disease patients in study 2. This increase is stronger in apathetic individuals. Error bars refer to one SE. See also Figures S2, S3, S4, and S7.

Consistent with our findings in PD, we found that healthy people were more fluent at generating path options after taking cabergoline compared to placebo (*t*(28) = 3.49; p = 0.002; BF_10_ = 21.9; Figure 4A). Yet they exhibited lower mean uniqueness in their paths (*t*(28) = −2.17; p < 0.05; BF_10_ = 1.49; Figure 4B) and were less explorative in the 2-dimensional path subspace (*t*(28) = −2.31; p < 0.05; BF_10_ = 1.90; Figure 4C). Thus increased levels of dopamine shifted behavior toward producing more options, at the expense of reduced uniqueness (Figure 4D). There was no significant effect of testing on repeated sessions (Figure S3).

As for the PD analysis, we corrected for the correlation between fluency and uniqueness using a mixed model. The effect of each path’s duration on uniqueness was quantified as a function of drug state and apathy. Similar to the effect of dopaminergic treatment in PD, the D_2_ agonist was found to increase the uniqueness of paths for a given fluency (Figure 4E; *t*(3,838) = 3.88; p < 0.001). Furthermore, in this study, there was a significant interaction between drug state and apathy. This indicated that the effect of cabergoline in increasing uniqueness for a given fluency was stronger in apathetic individuals (*F*(1, 3,824) = 16.4; p < 0.001; Figure S3).

**Figure 5:**
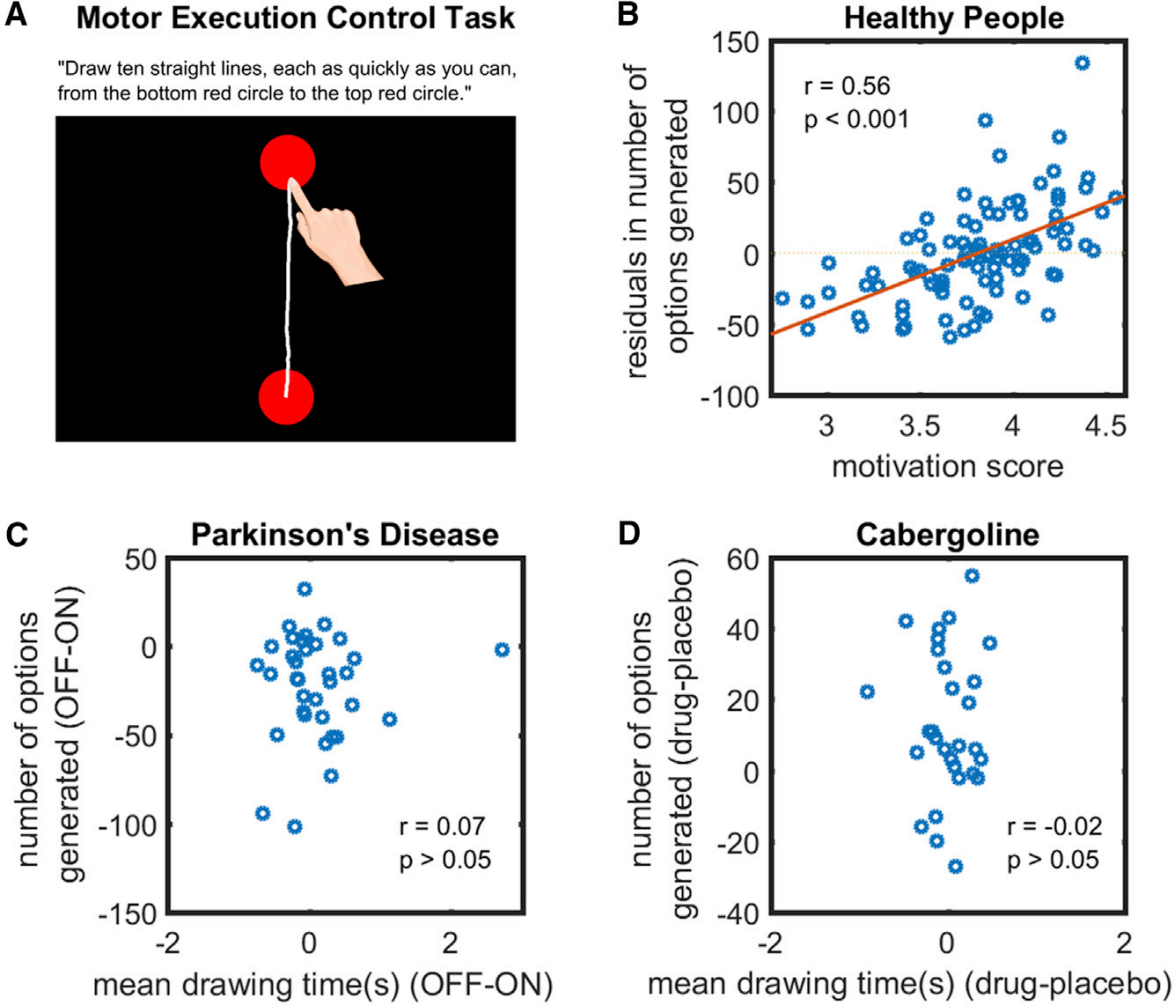
Results from Motor Execution Control Task (A) Participants had to draw ten straight lines, each as quickly as they could, from a start point (bottom circle) to an end goal (top circle). This serves as a baseline index of their motor vigor. (B) In healthy people (n = 96), the significant association between fluency in the option-generation task and individual level of motivation (reported above in Figure 2B) remains even after regressing out the mean time taken to draw each line. (C) In PD (n = 35), the difference in the number of paths generated for the option-generation task between the ON and OFF states did not correlate with their difference in mean drawing speed on the motor execution control task. (D) In healthy elderly people (n = 29), the difference in option generation fluency when on cabergoline and placebo also did not relate that in motor execution ability. These suggest that the reduced fluency in option generation when PD patients are OFF dopamine and when healthy people are on placebo is not attributable to impairments in movement speed or executing actions. See also Figure S6.

Our task quantifies option generation using behavioral output. However, as with traditional timed tests of fluency, several other factors may confound this interpretation. First, participants—including PD patients—might have generated path options, but not produced them because of deficits in motor execution, e.g., if the individual’s movements were generally slowed. To account for motor execution, we administered a motor execution control task *before* the option generation task in order to test baseline drawing speed (study 4; STAR Methods). Participants had to draw ten straight lines, each as quickly as they could, between the two fixed points of the option generation task (Figure 5A). The mean time taken to draw each line (excluding time between lines) served as a baseline index of their motor vigor that was closely matched to the main task.

**Figure 6:**
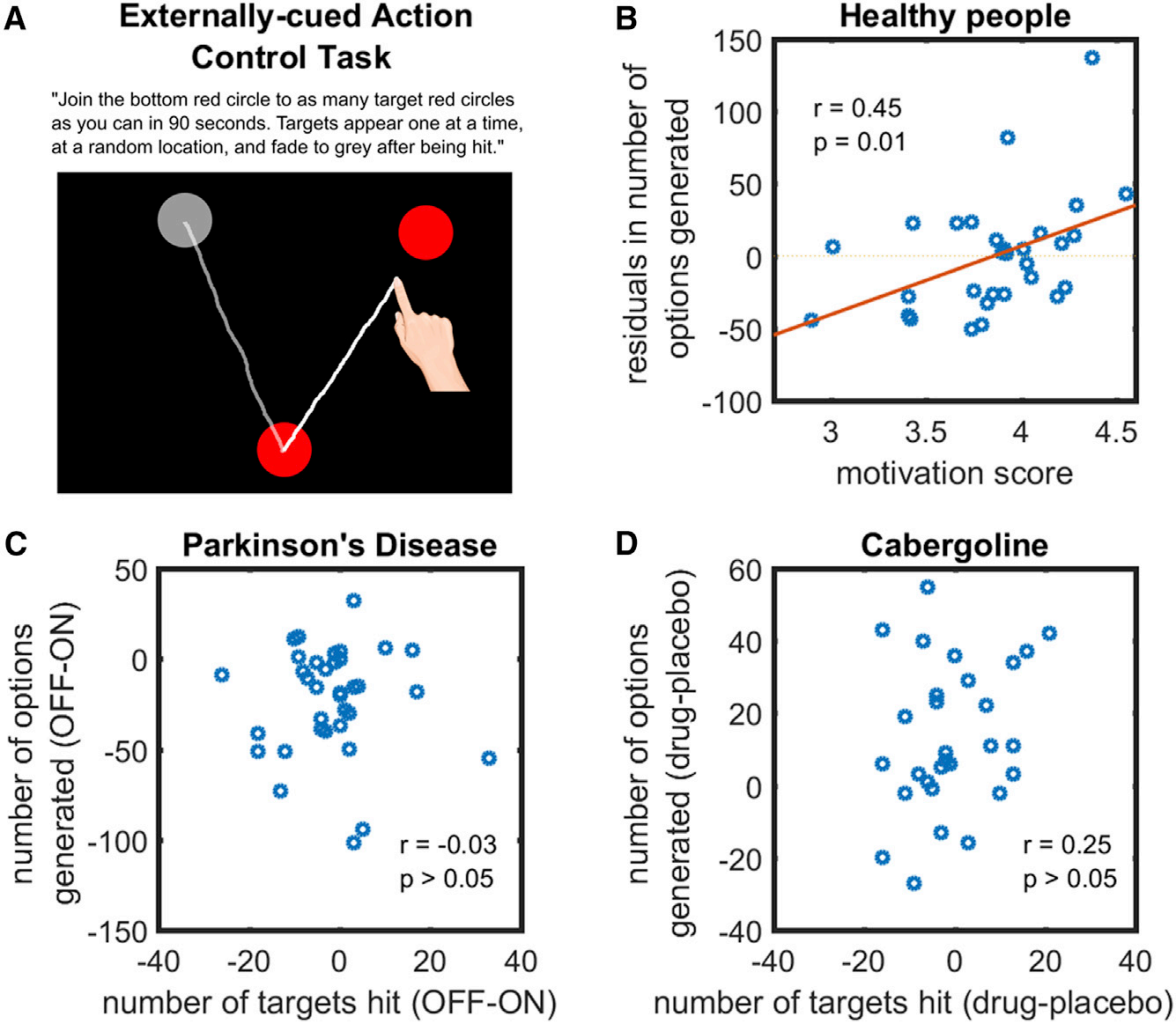
Results from Externally Cued Action Control Task (A) Participants were asked to join the bottom circle to as many target circles as possible in 90 s. Only one target, which was always red in color, appeared at a time. It turned gray after being touched, and then a new red target appeared. Thus, this task does not require either option generation or selection but requires motor planning of a different action each time. (B) In healthy people (n = 30), there was a significant correlation between fluency in the option-generation task and individual level of motivation even after regressing out the number of targets hit in this control task. (C) In PD (n = 35), the change in fluency in the option-generation task between the ON and OFF states did not relate to the change in the number of targets hit on the externally cued action control task. (D) In healthy elderly people (n = 29), the difference in option generation fluency when on cabergoline and placebo also did not relate that in motor planning ability. This indicates that reductions in fluency of option generation when PD patients are OFF dopamine and when healthy people are on placebo is not explained simply by deficits in planning or initiating actions. See also Figures S6 and S7.

A second challenge might be that, whereas participants might have generated options, they might encounter difficulty *planning* the motor actions required to execute them. To examine this, we employed an externally cued action control task (study 5; STAR Methods), in which each movement required a different motor plan, but there was no need to generate unique paths. Instead, participants simply had to draw a straight line to a target location that was randomly generated by the computer. A new target location was generated once the path had been completed, and the goal was to connect to as many target locations as possible in 90 s (Figure 6A). This allowed us to assess motor planning ability, in isolation from the ability to generate the option for the next action because this was now provided by the computer.

**Figure 7:**
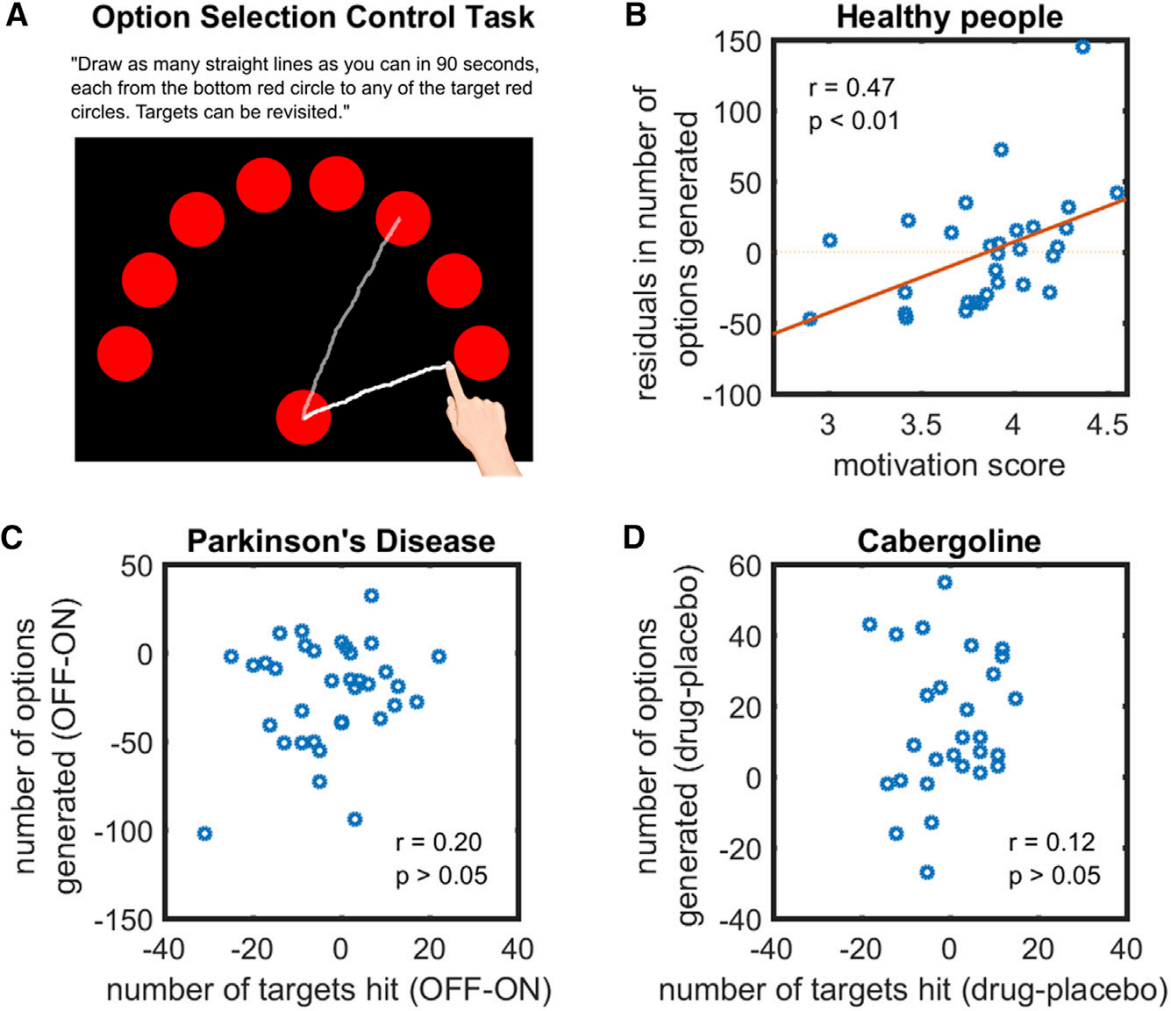
Results from Option Selection Control Task (A) Participants had to join the bottom circle to as many target circles as possible in 90 s. They saw an array of 24 possible targets on the screen and were free to select any target they wanted for each connection (only 8 targets are illustrated here for simplicity). Thus, this task required selection between options, but not the generation of possible options. (B) In healthy people (n = 30), fluency in the option-generation task correlated with individual level of motivation even after accounting for the ability to select options. (C) In PD (n = 35), the difference in number of paths generated on the option-generation task between the ON and OFF states does not associate with the difference in number of paths drawn on the option selection control task. (D) In healthy elderly people (n = 28), the difference in option generation fluency when on cabergoline and placebo also did not relate that in option selection ability. This suggests that reductions in fluency of option generation when PD patients are OFF dopamine and when healthy people are on placebo is not simply due to impairments in selecting among generated options. See also Figures S6 and S7.

Finally, it is possible that, whereas an individual might have generated many path options, they might have encountered difficulty in *selecting* from among them—a deficit in option selection during decision making. To examine this, we administered an option selection control task (STAR Methods) where participants had to first select an option from a given set of possible target locations and then draw a straight path to it. The goal was to draw as many individual paths as possible from a central start location in 90 s to the displayed possible target locations (Figure 7A). No unique generation of paths was required as the target options were always displayed.

In healthy people, performance on the three control tasks showed no significant relationship of drawing speed, motor planning, and option selection with fluency on the option generation task or level of motivation (Figure S6). Crucially, a significant correlation between fluency and motivation remained even after controlling for motor execution (r = 0.56; p < 0.001; BF_10_ > 100; n = 96; Figure 5B), motor planning (r = 0.45; p = 0.01; BF_10_ = 4.2; n = 30; Figure 6B), and option selection ability (r = 0.47; p < 0.01; BF_10_ = 6.0; n = 30; Figure 7B).

In PD (n = 35), the difference in fluency on the option generation task between ON and OFF states did not associate with that on the motor execution (r = 0.07; p > 0.05; BF_10_ = 0.23; Figure 5C), externally cued action (r = −0.03; p > 0.05; BF_10_ = 0.21; Figure 6C), and option selection control tasks (r = 0.20; p > 0.05; BF_10_ = 0.40; Figure 7C). The difference in option generation fluency between healthy elderly people (n = 29) when on cabergoline and on placebo also did not relate to the difference in motor execution (r = −0.02; p > 0.05; BF_10_ = 0.23; Figure 5D), motor planning (r = 0.25; p > 0.05; BF_10_ = 0.51; Figure 6D), and option selection ability (r = 0.12; p > 0.05; BF_10_ = 0.28; Figure 7D). Furthermore, in both PD patients and cabergoline participants, repeated-measures ANOVA found that dopamine’s improvement in fluency was specific to the option-generation task. There was no effect of dopamine on fluency in the externally cued action and option selection control tasks (Figure S7). Taken together, these results indicate that reductions in the number of options generated in the PD OFF or placebo states are not due to deficits in movement speed or executing actions, in planning or initiating actions, or in selecting among generated options.

To the best of our knowledge, this is the first study causally implicating dopamine as a key neuromodulator of self-generated options for behavior. The prefrontal cortex has long been identified as playing a role in executive functions that may be crucial for the initiation and sustaining of responses [5, 6]. Evidence from neuroimaging [7, 8], electrophysiological [9], patient [10, 11], and lesion [12, 13] studies have also implicated a key role for the frontal lobe in creative thinking. Together, these suggest that the prefrontal cortex plays an important role in producing options that are both numerous (fluent) and unique. The neurochemical basis of these two putative frontal lobe processes, however, was unknown. The findings presented here using two within-subject manipulation studies demonstrate that both PD patients and healthy people performing our option-generation task behave differently depending on the level of dopamine present. PD patients ON dopamine and healthy individuals on cabergoline generated a larger number of path options but at the expense of reduced creativity. They produced behavioral options of lower mean uniqueness and exhibited lesser variation in their generated options.

Our analyses uncovered two distinct effects of dopamine: it increased the fluency of generating options but diminished overall uniqueness due to a natural trade-off between fluency and uniqueness (Figures 2, 3, and 4). However, after correcting for this trade-off, dopamine was found to in fact increase uniqueness for a given fluency of option generation (Figure S3). Therefore, although the major effect of dopamine was to improve the fluency of producing options, it also independently improved uniqueness for a given fluency.

A recent theoretical perspective by Boot and colleagues [22] proposed that the balance of fronto-striatal dopamine levels might mediate the trade-off between the processes of flexibility and persistence. Striatal dopamine via the nigrostriatal pathway promotes flexible processing and facilitates original ideation (i.e., uniqueness), whereas prefrontal dopamine via the mesocortical pathway modulates persistence, leading to focused, systematic thinking within the same conceptual category (i.e., fluency). Although our findings are insufficient to verify the mechanisms proposed by this model, they do provide strong support for the premise that the balance between flexibility and persistence is indeed influenced by dopamine.

Several lines of research, e.g., genetic [16, 17], neuroimaging [18, 19], and patient [20, 21] studies, have suggested that dopamine improves creativity. On our task, dopamine decreases overall uniqueness due to its effects on fluency. However, after accounting for the fluency-uniqueness trade-off, we found a true increase in uniqueness—dopamine shifts the trade-off line so that participants are more unique for a given fluency. These two distinct effects of dopamine, on fluency and on uniqueness, were separable in our paradigm as it employs a fine-grained quantification of option uniqueness compared to many other tests.

The primary aim of our study was to understand the role of dopamine in option generation, but in the process, we also investigated whether motivation (or apathy) might be related to these variables. Highly motivated healthy individuals generated more options that were less unique (Figure 2), and the D2 agonist increased fluency without improving uniqueness. In apathetic healthy individuals, the D2 agonist improved both fluency and uniqueness for a given fluency of generation (Figure S3). Intriguingly, in PD, neither apathy nor its interaction with dopamine levels influenced the fluency or uniqueness of options generated (Figure S3; Table S3). This indicates that, whereas difficulty generating options might contribute to apathy in the healthy population, pathological apathy is likely to be influenced by other factors.

This is interesting because recent findings suggest that dopamine may play a key role in modulating apathy [15]—a disorder of motivation characterized by reductions in self-initiated actions [32]. Apathy can be profoundly disabling for patients with neurodegenerative conditions, including PD, and negatively impacts the healthy population to varying degrees [33]. Although apathy is commonly framed as a disorder of evaluating options, it seems to selectively impair internally generated action, and thus, impairments in the ability to self-generate possible options for action may contribute to a lack of motivation to act [15].

A recent study reported that apathy in PD correlates with sensitivity to the value of rewards being presented [34]. This appears to be consistent with results that demonstrate greater impairments in incentive processing in apathetic PD patients compared to non-apathetic patients and healthy controls [35]. Thus, the evaluation and selection of options during decision making based on their potential rewarding outcomes or the effort required to obtain them might be more important contributory factors to apathy in PD than inability to generate options for behavior [15, 34].

The neuroscience of self-generated behavioral choices is an exciting field that is still in its infancy. Uncovering key neurobiological components is crucial to advancing our understanding of decision making. Here, we have shown that dopamine is a key neurotransmitter involved in this process, modulating option generation along a uniqueness-fluency spectrum.

## STAR★Methods

### Key Resources Table

**Table:** 

REAGENT or RESOURCE	SOURCE	IDENTIFIER
**Software and Algorithms**		

MATLAB	MathWorks	https://www.mathworks.com
Custom-built MATLAB code	This paper	N/A
SPSS Statistics 22.0	IBM	https://www.ibm.com/uk-en/marketplace/spss-statistics
JASP	[36]	https://jasp-stats.org/

### Contact for Reagent and Resource Sharing

Further information and requests for resources and reagents should be directed to and will be fulfilled by the Lead Contact, Yuen-Siang Ang (yuensiang.ang@bnc.ox.ac.uk).

### Experimental Model and Subject Details

#### Participants

96 healthy people took part in study 1. The sample comprised 60 young participants recruited from the Oxford Psychology Research participant recruitment scheme (31 males, 29 females; mean age = 24.8, SD = 4.7) and 36 older people (22 males, 14 females; mean age = 70.3, SD = 6.8) from the Oxford Dementia and Aging Research database. All participants had corrected-to-normal vision, no history of psychiatric or neurological conditions and were paid a fixed rate for participating. They gave written informed consent and the study was approved by the University of Oxford ethics committee.

35 patients with PD (23 males, 12 females; mean age = 67.7, SD = 8.1) and 34 healthy age-matched controls (21 males, 13 females; mean age = 69.1, SD = 8.3) participated in study 2. They gave written informed consent and the study was approved by the University of Oxford ethics committee. All patients were recruited from clinics in the Oxfordshire area, had a clinical diagnosis of idiopathic PD according to Queen Square Brain Bank criteria and no history of other major neurological or psychiatric conditions. They were established on levodopa therapy. 15 patients took this as their sole PD medication, 11 were on a concomitant dopamine agonist, and a minority were on other adjunctive therapies (Monoamine oxidase inhibitor N = 8; Amantadine N = 5; anti-cholinergic N = 1). Demographics, levodopa equivalent dose and UPDRS are presented in Table S1. They were tested in two counter-balanced sessions, once after having taken their dopaminergic medication as usual (‘ON’) and once after overnight withdrawal > 12h (‘OFF’). All healthy controls were recruited from a volunteer database, had corrected-to-normal color vision and no history of psychiatric or neurological conditions. 18 controls were tested in a single session while 16 were tested twice across two sessions.

29 healthy elderly people (18 males, 11 females; mean age = 68.4, SD = 4.2) recruited from the Oxford Dementia and Aging Research database took part in study 3. They had corrected-to-normal vision, no history of psychiatric or neurological conditions and were paid a fixed rate for participating. All participants gave written informed consent and the study was approved by the University of Oxford ethics committee.

### Method Details

#### Experimental setup

The experiment was carried out in a dimly-lit quiet room. All tasks were programmed with PsychToolBox on MATLAB (MathWorks) and implemented on a 23” touchscreen computer (model: Dell P2314T), at screen resolution 1920 × 1080 at 60Hz frame rate, width 509 mm and height 286 mm. The touchscreen was placed vertically upright at a viewing distance of ∼50 cm, and the monitor’s height and angle of tilt was adjusted to each participant’s comfort. Importantly in study 1, the participant was left alone in the testing room for the option generation task to prevent performance anxiety.

#### Option generation task

In this task, two red circles were displayed vertically at the center of a touchscreen computer, separated by a distance of 204 mm (Figure 1A). Each circle had a radius of 13 mm. Participants were instructed to “Draw as many different paths as you can from the bottom red circle to the top red circle in 4 minutes.” Real-time visual feedback was provided such that paths appeared as participants drew them, regardless of the starting point of the movement. Drawn paths were allowed to intersect, and remained on the screen during the whole task so load on working memory was minimized. Paths were counted as valid as long as they started and ended within 21 mm of the centers of the bottom and top circles respectively. This allowed for an error margin in finger movements.

#### Motor execution control task

This task was administered *before* the option generation task in order to test baseline drawing speed. Participants saw two red circles on the touchscreen (as in the option generation task), but were instructed to “Draw ten straight lines, each as quickly as you can, from the bottom red circle to the top red circle” (Figure 5A). Real-time visual feedback was provided such that paths appeared as they were drawn, but lines were erased from the screen between movements. The mean time taken to draw each line (excluding time between lines) served as a baseline index of their motor vigour that was closely matched to the option generation task.

#### Externally-cued action control task

In this task, participants were required to “Join the bottom red circle to as many target red circles as you can in 90 seconds. Targets appear one at a time, at a random location, and fade to grey after being hit” (Figure 6A). The starting point (bottom red circle) always remained fixed, at the same position as in the option generation task. Unknown to participants, targets were always equidistant from, but at a random angle to, the starting point. This distance was the same as the two red circles in the option generation task (i.e., 204 mm). The range of angles from the starting point to the target was ± 90 degrees and real-time visual feedback was provided. This task and the option selection control task were administered in a counterbalanced order after the option generation task.

#### Option selection control task

In this task, participants were presented with an array of 24 red targets equally spaced in an arc, each 204 mm in distance from the starting point (i.e., red bottom circle). They were required to “Draw as many straight lines as you can from the bottom red circle to any target red circle in 90 seconds. Targets can be revisited” (Figure 7A). Real-time visual feedback was provided as in the option generation task.

#### External measures

In study 1, we assessed level of motivation with a modified version of the Lille Apathy Rating Scale (LARS). The LARS is a clinical interview that assesses apathy (or the lack of motivation) based on the patient’s life over the past four week. Four domains are measured: intellectual curiosity, action initiation, self-awareness, and emotions [37]. To create a comparable measure suitable for the general population, a team of clinical neurologists and university researchers developed, based on their experience with clinically apathetic patients, novel items to specifically reflect each domain of the LARS. Items from the clinical LARS that were deemed to be applicable to healthy people were also adapted. This gave rise to the modified LARS, a 51-item self-report questionnaire of motivation that has been validated in the healthy population [38]. Participants had to rate from 1 to 5 how true (1: completely untrue; 5: completely true) each item was based on the past two weeks of his life (e.g., “I was easily able to decide to do things by myself, without needing someone to push or encourage me”). The higher the mean modified LARS score, the higher the level of motivation.

In study 2, participants completed the LARS to measure levels of apathy-motivation. The LARS has a total score ranging from −36 to +36, with a higher score indicating lower levels of motivation. To make the interpretation of scores across the LARS and modified LARS consistent, we reversed the signs of all scores obtained from the LARS so that a higher score indicates greater motivation (and lower apathy) instead. Participants were screened for clinical depression based on the Beck’s Depression Inventory (BDI) [39] and their cognitive ability was assessed with Addenbrooke’s Cognitive Examination (ACE-III) [40]. Patients had significantly lower ACE scores and significantly higher BDI scores compared to the healthy controls; however, the average scores in both groups did not meet the cut-off for suspected dementia (ACE-III < 82) nor depression (BDI > 20). PD patients also completed the Unified PD Rating Scale (UPDRS) to assess disease severity. Details of all demographics can be found in the Table S1.

In study 3, participants completed the LARS and ACE-III. Details of these demographics are available in Table S2**.**

#### Metric of similarity between traced paths

Each path *i* was represented as a set of coordinates $\;x_{i}\left(t\right),y_{i}\left(t\right)$, for each time step of the path. First, the coordinates along each path were re-sampled at 200 points along the path, as a function of distance along the path. To do this, distance along the path was calculated as $s_{i}\left(t\right)=\sum_{\tau =1}^{t}\left|\left[\begin{array}{c} x_{i}\left(\tau \right)-x_{i}\left(\tau -1\right)\\ y_{i}\left(\tau \right)-y_{i}\left(\tau -1\right) \end{array}\right]\right|$, and a new vector of coordinates $h_{i}\left(s\right)$ was calculated using linear interpolation along s(t). The feature vector $h_{i}\left(s\right)$ thus describes the trajectory of path *i* with 400 numbers, in a uniform way across different paths drawn. This enables different paths to be compared. In order to include other characteristics of the paths, we included extra features in addition to the screen position, to capture other features of the trajectory’s shape. The first derivative of the path direction $\dot{h}=h\left(s\right)-h\left(s-1\right)$ and the second derivative $\ddot{h}$ were calculated. These values were averaged for bins of 10 distance units, to give vectors 20 values over the path. Thus in total, the feature vector v for each path contained 480 values, with $v_{i}=\left[h_{x},h_{y}{\dot{h}}_{x},{\dot{h}}_{y},{\ddot{h}}_{x},{\ddot{h}}_{\text{y}}\right]$.

To estimate how different any pair of paths were from one another, we subtracted the features of one path from the other to give $d_{ij}=\left|v_{i}-v_{j}\right|$. To account for left-right mirror similarity, the difference $d_{ij}^{'}$ was also calculated using the mirror-image path (i.e., with $h_{x}\to -h_{x}$, and the related derivatives), and the minimum value of $d_{ij}$ and $d_{ij}^{'}$ was used.

The difference metric between any pair of paths enabled the uniqueness of a path to be calculated, as the smallest difference from any other path that was generated by any participant in all three studies, $u_{i}=\underset{j}{\min}\left(d_{ij}\right)\;\left[i\neq j\right]\;$. If $u_{i}$ is large, the path is not similar to any other paths.

To visualize the paths of a single participant in a 2-dimensional space, metric multidimensional scaling was performed on the dissimilarity matrix $d_{ij}$. The default fitting algorithm using a metric stress criterion was used from the Statistics Toolbox in MATLAB. This algorithm assigns a two-dimensional “coordinate” to each path, such that the difference metric between each pair of paths matches (as closely as possible) the distance between a point in a 2-dimensional plane.

Our similarity metric to quantify how dissimilar generated paths were to each other necessarily makes assumptions and simplifications, but the method used here has several strengths:

1) The metric is insensitive to whether the same path is drawn slower or faster, yet it is sensitive to the order in which places are visited.
2) Changes in direction are given extra weight due to inclusion of derivatives, giving special considerations to distinguishing perceptually salient differences like sharp corners or smooth curves.
3) The metric is insensitive to small deviations at any point on the route, but large deviations are emphasized due to the 2-norm and because we parameterise along the path.

Different norms (such as manhattan distance) and many more sophisticated algorithms are surely possible, but we argue that our method is relatively transparent and assumption-free. Furthermore, we also examined uniqueness using 1-norm and 3-norm distances and found that they give the same result, strongly correlating with uniqueness from our original 2-norm metric (*r*_1-norm_ = 0.996, p < 0.001; *r*_3-norm_ = 0.998, p < 0.001).

### Quantification and Statistical Analyses

The behavioral data was analyzed using MATLAB and IBM SPSS Statistics 22.0. We tested the assumption of normality in all data, and those that were not normally distributed were log-transformed before further analyses. Dependent variables were compared within-subjects using a paired two-tailed t test and between-subjects using an independent-samples two-tailed t test. For all analyses, a *p-value* < 0.05 was taken to be statistically significant unless otherwise stated. Bayesian statistical analyses for correlations and t tests were also conducted using JASP [36] in order to complement classical statistics. The Bayes Factor (BF_10_) quantifies the amount of evidence in favor of the alternative hypothesis (H_1_) and generally [41]: 1 < BF_10_ < 3 indicates anecdotal evidence, 3 < BF_10_ < 10 indicates substantial evidence, 10 < BF_10_ < 30 indicates strong evidence, 30 < BF_10_ < 100 indicates very strong evidence, and BF_10_ > 100 indicates extreme evidence for H_1_.

### Data and Software Availability

Dataset and custom-built MATLAB code can be requested directly from the Lead Contact.

## Supplemental Information

Document S1. Figures S1–S7 and Tables S1–S3

Document S2. Article plus Supplemental Information
