# Supplementary material for: Dopamine Modulates Option Generation for Behavior
Source: Curr Biol. 2018 May 21;28(10):1561–1569.e3. doi: 10.1016/j.cub.2018.03.069 (PMC5981001; doi:10.1016/j.cub.2018.03.069)
Supplement: Document S1. Figures S1–S7 and Tables S1–S3 [file mmc1.pdf]

Current Biology, Volume *28*

Supplemental Information

Dopamine Modulates Option Generation for Behavior

Yuen-Siang Ang, Sanjay Manohar, Olivia Plant, Annika Kienast, Campbell Le Heron, Kinan Muhammed, Michele Hu, and Masud Husain

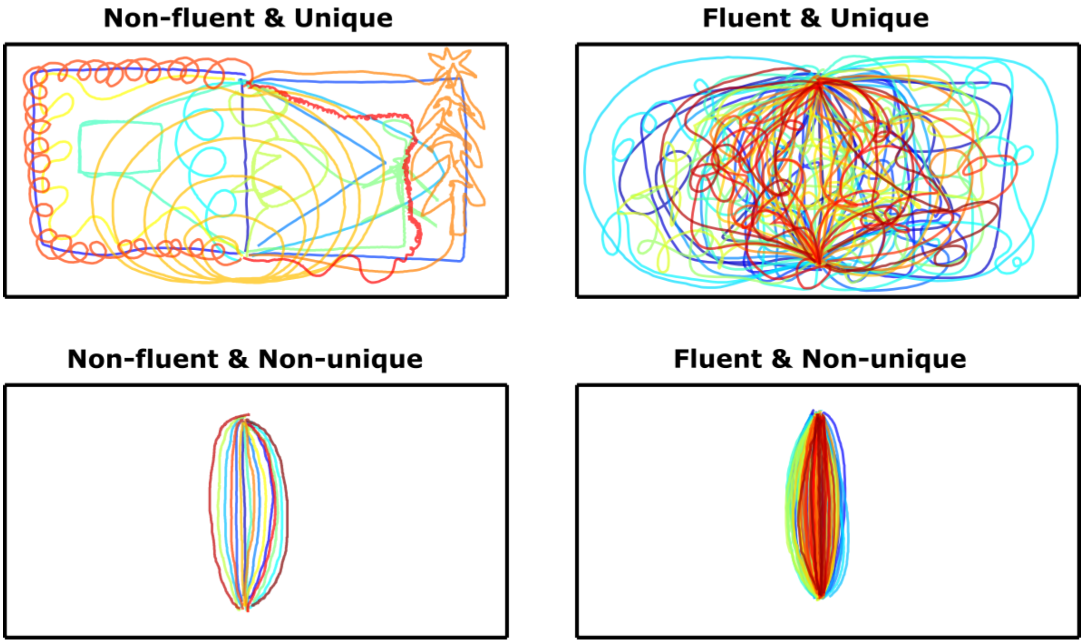

Figure S1. Examples of path options produced by individuals (in studies 1–3) that were (i) non-fluent and unique, (ii) fluent and unique, (iii) non-fluent and non-unique, and (iv) fluent and non-unique. Related to Figure 1.

Although participants tended to either produce many similar paths or came up with fewer unique paths, generating more paths is not necessarily associated with reduced uniqueness (and vice versa). Some individuals were both fluent and unique, and some non-fluent and non-unique. We defined <25th percentile as the cut-off for being non-fluent and non-unique and >75th percentile as that for being fluent and unique.

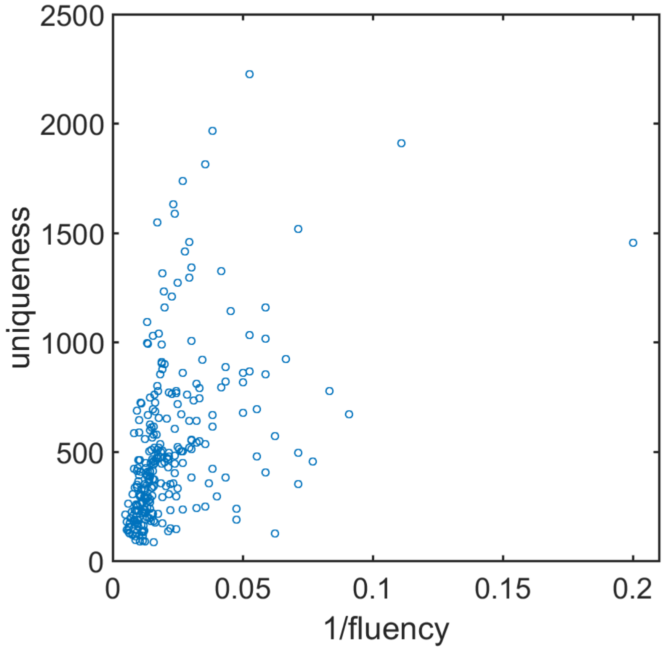

Figure S2. Scatterplot of subjects' mean uniqueness against inverse of their fluency (or path duration) for all three studies. Related to Figure 3F and 4E.

A direct manner to examine option generation in our task might be a summation of uniqueness over all paths in order to first obtain a global score, before breaking down into fluency and uniqueness. This would account for the fact that generating two paths with *x* uniqueness in 10 seconds is equivalent to generating one path with *2x* uniqueness in 10 seconds. However, this assumes that fluency and uniqueness are linearly related, which is not the case in the task. In other words, a unit change in fluency does not equate to a unit change of uniqueness. As such, we decided to adopt a linear mixed effects model analysis as described in Figure S3 instead.

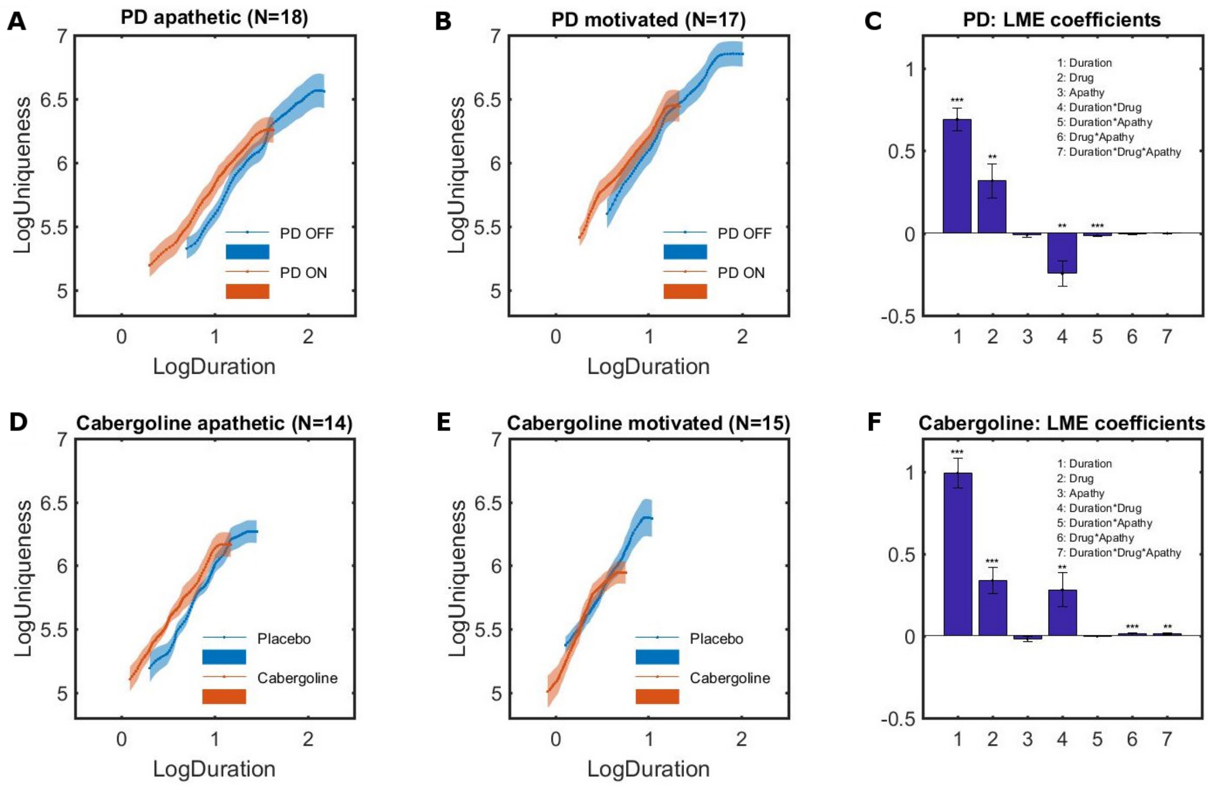

Figure S3. Effect of path duration on uniqueness as a function of drug-state and apathy (studies 2 and 3), accounting for mirror-image routes. Related to Figure 3F and 4E.

Because fluency correlates with uniqueness, a linear mixed-effects model for the uniqueness of each path produced by the PD patients in study 2 and healthy people in study 3 was fitted by maximal likelihood using the *fitlme* function from the Statistics Toolbox in MATLAB 2017a. We used fixed effects of each path's duration, dopamine drug state, apathy level and their interactions, and random effects of subject:

Uniqueness ~ Duration + Drug + Apathy + Duration*Drug + Duration*Apathy + Drug*Apathy + Duration*Drug*Apathy + (1|Subject)

In PD, the fixed effect terms of Duration and Drug were significant, indicating that dopamine increases uniqueness when controlling for duration. In other words, given the same duration of drawing time, PD patients when ON dopamine were more likely to generate a path of greater uniqueness than when OFF dopamine. There were also weaker but significant interaction effects of Duration*Drug and Duration*Apathy, indicating that drug and apathy

changes the slope of the speed-uniqueness trade-off. [Duration: $t(3661)=9.42$, $p<0.001$; Drug: $t(3661)=2.81$, $p=0.005$; Apathy: $t(3661)=-0.77$, $p>0.05$; Duration*Drug: $t(3661)=-2.91$, $p=0.004$; Duration*Apathy: $t(3661)=-5.67$, $p<0.001$; Drug*Apathy: $t(3661)=-1.26$, $p>0.05$; Duration*Drug*Apathy: $t(3661)=-0.29$, $p>0.05$].

In the cabergoline study, there were significant fixed effects of Duration and Drug, showing that healthy people were likely to generate a more unique path when on cabergoline compared to placebo. The interaction term of Drug*Apathy was also significant, indicating that the effect of cabergoline in increasing uniqueness for a given speed was stronger in apathetic individuals. Finally, there were also significant interaction terms of Duration*Drug and Duration*Drug*Apathy. [Duration: $t(3838)=14.1$, $p<0.001$; Drug: $t(3838)=3.88$, $p<0.001$; Apathy: $t(3838)=-0.66$, $p>0.05$; Duration*Drug: $t(3838)=3.27$, $p=0.001$; Duration*Apathy: $t(3838)=1.44$, $p>0.05$; Drug*Apathy: $t(3838)=3.81$, $p<0.001$; Duration*Drug*Apathy: $t(3838)=3.23$, $p=0.001$].

To plot the graphs, for each subject the uniqueness of their generated routes was taken in a sliding window calculated on the route durations. The mean log uniqueness in each of 80 overlapping windows (0.2 quantile width) was averaged across subjects, and plotted against the mean log duration for that window. This was performed for each drug condition separately. Shaded area represents standard error of the mean across subjects.

We did not find any significant effect of testing on repeated sessions in PD patients ($N=35$). A repeated measures ANOVA analysis found only a significant main effect of drug (ON, OFF), but not session nor drug*session interaction on (i) the number of paths generated [drug: $F(1,66)=22.6$, $p<0.001$; session: $F(1,66)=1.45$, $p>0.05$; drug*session: $F(1,66)=0.07$, $p>0.05$], (ii) mean uniqueness of generated options [drug: $F(1,66)=15.8$, $p<0.001$; session: $F(1,66)=1.29$, $p>0.05$; drug*session: $F(1,66)=0.16$, $p>0.05$], and (iii) area of exploration in the 2-dimensional path subspace [drug: $F(1,66)=4.71$, $p<0.05$; session: $F(1,66)=0.03$, $p>0.05$; drug*session: $F(1,66)=0.08$, $p>0.05$]. For the healthy age-matched controls ($N=16$), there was also no significant difference across sessions in number of paths generated ($t(15)=-1.68$, $p>0.05$), or mean uniqueness ($t(15)=2.06$, $p>0.05$), or area of exploration in the 2-dimensional path subspace ($t(15)=1.95$, $p>0.05$).

We did not find any significant effect of testing on repeated sessions in healthy people on cabergoline($N=29$). A repeated measures ANOVA analysis found only a significant main

effect of drug (ON, OFF), but not session nor drug*session interaction on (i) the number of paths generated [drug: $F(1,54)=12.6$, $p<0.001$; session: $F(1,54)=0.26$, $p>0.05$; drug*session: $F(1,54)=0.009$, $p>0.05$], and (ii) mean uniqueness of generated options [drug: $F(1,54)=4.89$, $p<0.05$; session: $F(1,54)=0.04$, $p>0.05$; drug*session: $F(1,66)=3.44$, $p>0.05$]. For (iii) area of exploration in the 2-dimensional path subspace, the interaction effect was also significant [drug: $F(1,54)=5.48$, $p<0.05$; session: $F(1,54)=0.06$, $p>0.05$; drug*session: $F(1,54)=5.75$, $p<0.05$].

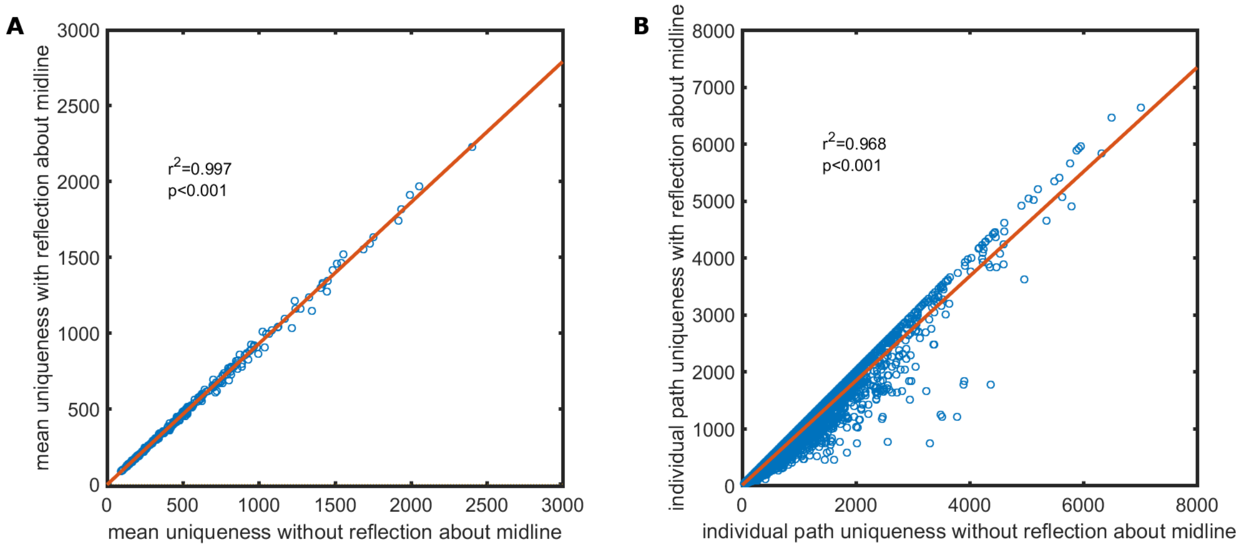

Figure S4. Comparison of mean subject uniqueness and individual path uniqueness between correcting and not correcting for mirror image paths. Related to Figure 3 and 4.

Although mirror image paths were considered as being similar to each other in external space, they require a considerably different motor program and thus, may be dissimilar in joint/muscle space. All of the analyses reported in the paper were re-ran using a distance metric that was not corrected for mirroring. Interestingly the uniqueness scores changed very little indeed, indicating that mirror images contributed only a small amount to uniqueness. In line with this, the *p*-values were very similar and do not affect interpretation of our results. The results of the linear mixed effects model analysis without accounting for mirror image paths are described below.

In PD, the fixed effect terms of Duration and Drug were significant, indicating that dopamine increases uniqueness when controlling for duration. In other words, given the same duration of drawing time, PD patients when ON dopamine were more likely to generate a path of greater uniqueness than when OFF dopamine. There were also weaker but significant interaction effects of Duration*Drug and Duration*Apathy, indicating that drug and apathy changes the slope of the speed-uniqueness trade-off. [Duration: $t(3661)=9.72$, $p<0.001$; Drug: $t(3661)=3.09$, $p=0.002$; Apathy: $t(3661)=-0.99$, $p>0.05$; Duration*Drug: $t(3661)=-3.15$, $p=0.002$; Duration*Apathy: $t(3661)=-5.13$, $p<0.001$; Drug*Apathy: $t(3661)=-0.91$, $p>0.05$; Duration*Drug*Apathy: $t(3661)=-0.55$, $p>0.05$].

In the cabergoline study, there were significant fixed effects of Duration and Drug, showing that healthy people were likely to generate a more unique path when on cabergoline compared to placebo. The interaction term of Drug*Apathy was also significant, indicating that the effect of cabergoline in increasing uniqueness for a given speed was stronger in apathetic individuals. Finally, there were also significant interaction terms of Duration*Drug and Duration*Drug*Apathy. [Duration: $t(3838)=11.0$, $p<0.001$; Drug: $t(3838)=4.27$, $p<0.001$; Apathy: $t(3838)=-1.10$, $p>0.05$; Duration*Drug: $t(3838)=2.72$, $p=0.007$; Duration*Apathy: $t(3838)=-0.34$, $p>0.05$; Drug*Apathy: $t(3838)=4.05$, $p<0.001$; Duration*Drug*Apathy: $t(3838)=2.85$, $p=0.004$].

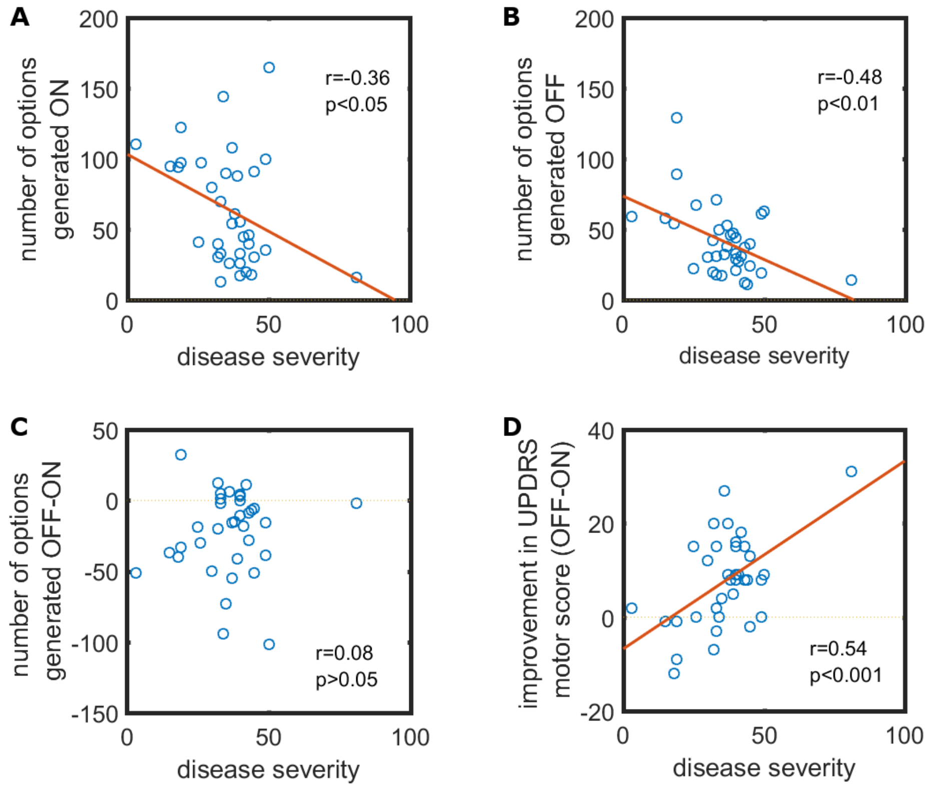

Figure S5. Correlation plots for disease severity on option generation. Related to Figure 3.

Could the effect of dopamine on increasing fluency be simply due to improvement in the motor deficits that are characteristic of PD? This is unlikely. **(A, B)** Disease severity was assessed using section III of the Unified Parkinson's Disease Rating Scale (UPDRS) when the patient was OFF dopamine. The fluency of generation correlated negatively with disease severity both ON and OFF dopamine, suggesting that the severity of disease modulated baseline number of paths generated. **(C)** Importantly, disease severity did *not* correlate with the difference in fluency between the ON and OFF states. **(D)** The *difference* in UPDRS motor scores between the ON and OFF states correlated strongly with that in the OFF state. This indicates that patients with worse PD severity also showed greater improvement in motor symptoms when treated with dopamine. These results show that disease severity modulates the baseline number of paths generated but has no effect on the dopaminergic improvement in fluency, despite strongly predicting improvement in motor UPDRS scores when on dopaminergic drugs.

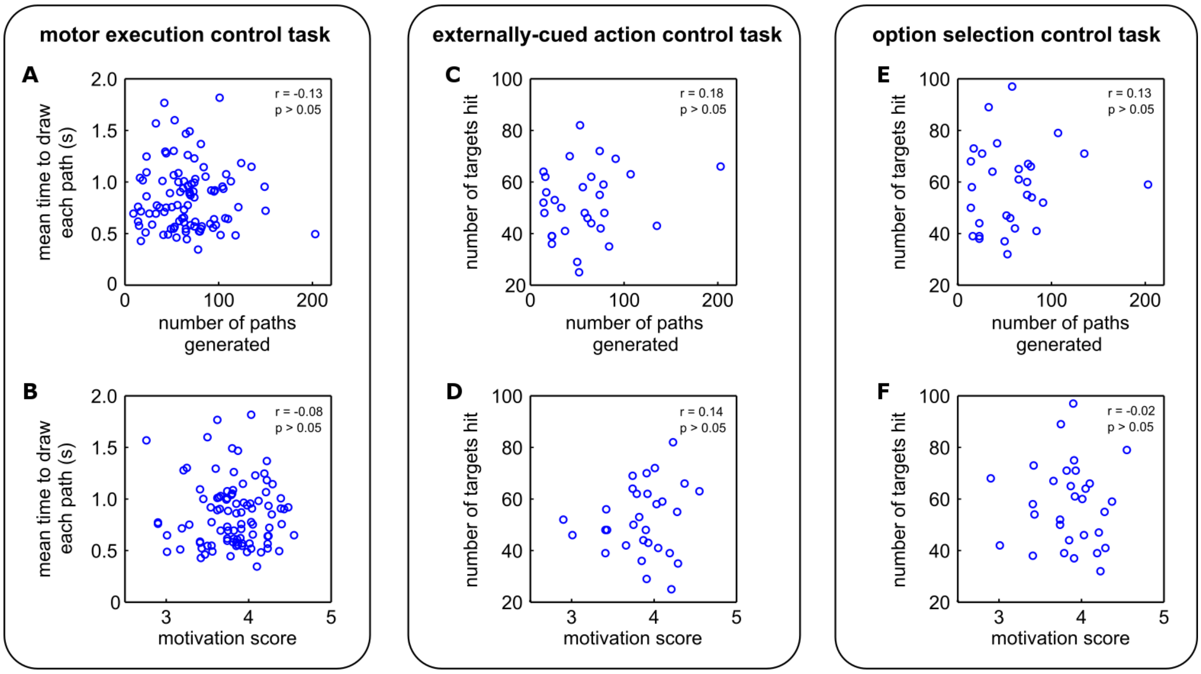

Figure S6. Correlation plots between control task performance, fluency of option generation and motivation in healthy people. Related to Figure 5B, 6B and 7B.

(A) There was no association between the mean time taken to draw each line on the motor execution control task and fluency in the option generation task, suggesting performance on the option generation task was independent of drawing speed. **(B)** There was also no significant correlation between the baseline drawing speed and individual level of motivation. (**C**) The number of targets hit in the externally-cued action control task did not relate to the number of paths generated, indicating that action planning did not influence performance on the option generation task. **(D)** There was also no significant correlation between the number of targets hit in this task and individual level of motivation. **(E)** Performance in the option selection control task was not related to fluency in the option generation task, suggesting that participants generated fewer paths not because of impairment in option selection. **(F)** There was also no significant correlation between performance on this task and individual level of motivation.

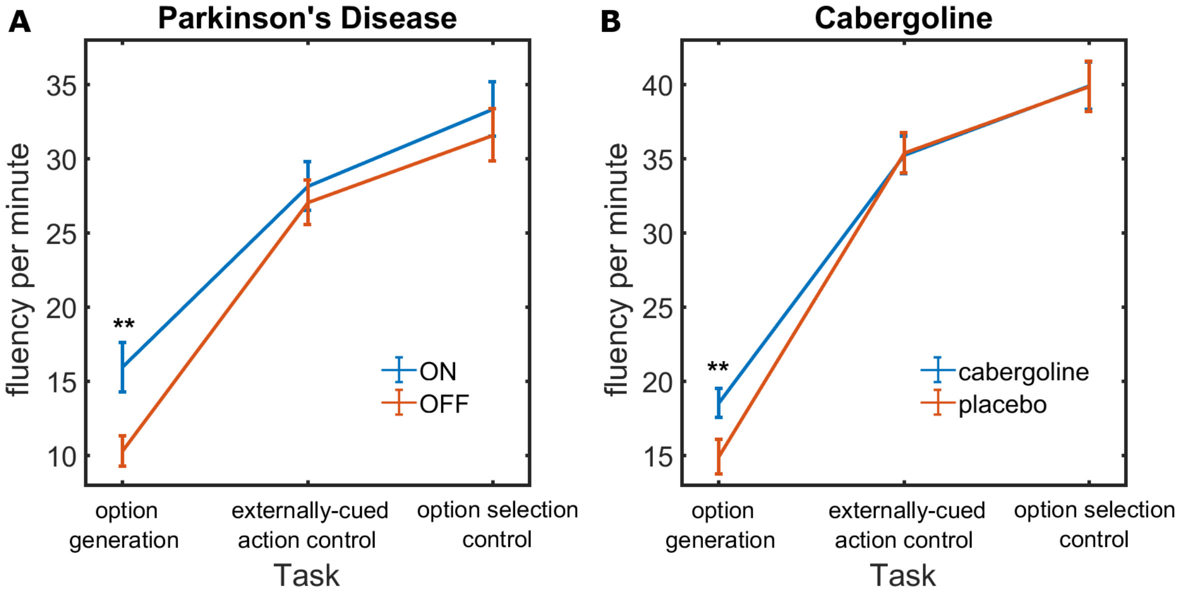

Figure S7. Comparison of fluency per minute for different tasks in (A) Parkinson's disease patients (study 2) and (B) healthy participants on cabergoline (study 3). Related to Figure 3, 4, 6 and 7.

A repeated measures ANOVA was performed using within-subject factors of drug status (on, off) and task type (option generation task, option selection control task, externally-cued action control task). There was a significant interaction effect (PD: $F(2,68)=4.66$, $p=0.01$; cabergoline: $F(2,54)=4.74$, $p=0.01$). Post-hoc comparison tests revealed that dopamine significantly improved fluency on the option generation task (PD: $p<0.001$; cabergoline: $p<0.001$), but drug status did not have any effect on the option selection (PD: $p=0.20$; cabergoline: $p=0.97$) and externally-cued action (PD: $p=0.37$; cabergoline: $p=0.89$) control tasks. This strongly suggests that the improvement from dopamine is specific to option generation. Error bars refer to one standard error.

Table S1. Demographic details for PD patients and controls in study 2. Related to STAR Methods.

Variable	Healthy elderly controls	Parkinson's disease patients	Controls versus Patients *p*-value
N	34	35	n/a
Age (years)	69.1 (±8.3)	67.7 (±8.1)	0.49
Apathy-Motivation (LARS)	26.1 (±4.5)	21.1 (±7.4)	0.001*
Depression score (BDI)	3.5 (±3.0)	14.0 (±7.8)	<0.001*
ACE	95.5 (±4.5)	91.8 (±5.9)	<0.01*
UPDRS III ON	n/a	28.3 (±11.6)	n/a
UPDRS III OFF	n/a	36.3 (±12.5)	n/a
Levodopa equivalent dose (mg/24h)	n/a	623.0 (±359.2)	n/a

*: significant result

Note: LARS has been reversed here so that higher scores indicate greater motivation and lower apathy.

Table S2. Demographic details for healthy elderly participants in study 3. Related to STAR Methods.

**Variable**	
N	29
Age (years)	68.4 (±4.2)
Apathy-Motivation (LARS)	21.0 (±5.2)
ACE	97.4 (±2.8)

Note: LARS has been reversed here so that higher scores indicate greater motivation and lower apathy.

Table S3. Apathy did not relate to fluency or creativity in PD patients (study 2). Related to Figure 3.

	Number of paths generated			Mean uniqueness			Area of exploration in 2-dimensional subspace		
	**ON**	**OFF**	**ON – OFF**	**ON**	**OFF**	**ON – OFF**	**ON**	**OFF**	**ON – OFF**
**LARS total**	0.11	0.09	0.07	0.33	0.25	0.02	0.11	0.27	0.30

The total score on the LARS clinical interview (our measure of apathy-motivation) did not correlate with number of paths generated, or mean uniqueness, or area explored in path subspace in the ON and OFF states nor with the difference between the two states (all $p>0.05$, see **Table S3**). These findings suggest that apathy in PD was not related to fluency of generation or creativity. We then divided these patients into two groups – apathetic ($N=18$) and motivated ($N=17$) – based on our LARS apathy cut-off score of <22. A two-way mixed ANOVA analysis revealed a significant effect of drug (ON, OFF), but not apathy, nor drug*apathy on (i) the number of paths generated [drug: $F(1,33)=20.4$, $p<0.001$; apathy: $F(1,33)=0.65$, $p>0.05$; drug*apathy: $F(1,33)=0.70$, $p>0.05$], (ii) mean uniqueness [drug: $F(1,33)=13.8$, $p<0.01$; apathy: $F(1,33)=1.91$, $p>0.05$; drug*apathy: $F(1,33)=0.09$, $p>0.05$], and (iii) variation in paths [drug: $F(1,33)=5.08$, $p<0.05$; apathy: $F(1,33)=0.24$, $p>0.05$; drug*apathy: $F(1,33)=1.45$, $p>0.05$]. These findings suggest that unlike the healthy population, apathy in PD was not related to fluency of generation or creativity.
